# Demographic factors that impact attitudes toward medical cannabis

**DOI:** 10.1016/j.pecinn.2022.100085

**Published:** 2022-09-14

**Authors:** Thomas A. Clobes, Matin Gagnon

**Affiliations:** California State University Channel Islands, One University Dr., Camarillo, CA 93012, USA

**Keywords:** Medical cannabis, Health policy, Health education, Politics, Religion, Stigma

## Abstract

**Objective:**

This study aims to identify pertinent demographic characteristics that influence attitudes toward medical cannabis.

**Methods:**

Survey respondents were recruited through social media posts, partnering with community organizations, and snowball sampling. Attitudes were measured with a modified version of the medical component of the Recreational and Medical Cannabis Attitudes Scale (MMCAS). Data were analyzed using a one-way ANOVA or one-way Welch ANOVA to determine differences within demographic characteristics. A Tukey-Kramer, or Games-Howell, post-hoc analysis was conducted to determine specific groups within the independent variables that significantly impacted medical cannabis attitudes.

**Results:**

A total of 645 participants completed the survey. Significant variation in MMCAS was noted between groups based on race, political party affiliation, political view, religion, state legal status, and past/current cannabis use. There were no significant variations noted in MMCAS for apolitical factors.

**Conclusion:**

Political, religious, and legal demographic factors impact attitudes toward medical cannabis.

**Innovation:**

The use of health education targeted at the groups of people who continue to harbor antiquated attitudes toward medical cannabis will help to improve patient access and, thus, patient outcomes. Cannabis advocates can innovatively apply health education efforts to groups of people who are aligned with the demographic factors identified in this current work.

## Highlights

• Identifies demographic variables that shape attitudes toward medical cannabis
• Political, religious, and legal factors significantly associated with variations in attitudes.
• Health education should target groups most likely to have negative views toward medical cannabis.

## 1 Introduction

Cannabis approval ratings in the United States have fluctuated over time, with recent decades showing a large swing in favor of its legalization [1]. Prior to the 20th century, cannabis could be found as a medical treatment for melancholy and nervousness, among other ailments, though recreational use was not commonplace [2]. The first attempt at regulation came in 1906 when the Pure Food and Drug Act was passed. The Act required that cannabis be listed as an ingredient in products, but did not criminalize it [3]. The initial campaign to criminalize cannabis began in the 1920s, riding on the coattails of alcohol prohibition. Importantly, during this campaign, cannabis was placed in the same category as opiates and narcotics. By 1925, twenty-six states had passed legislation explicitly prohibiting the plant. The depictions of cannabis during this time were largely embellished by journalists and served as a century long basis for misinformation. The 1937 Marijuana Tax Act was the first federal legislation directly addressing cannabis individually [4]. The Act attempted to regulate cannabis by requiring dealers to pay a transfer tax. Ideologies surrounding cannabis remained negative with little distinction being drawn between other illicit drugs until the 1960s.

Later on, the War in Vietnam created rifts in American society, and cannabis became associated with the movement for peace [5,6]. However, even though public opinions on cannabis began to change dramatically, legislation remained staunchly opposed to its cultivation and consumption. The Controlled Substances Act of 1971 officially placed cannabis on a Schedule I status, deeming it highly addictive and having no medical purposes . Consistent with previous legislation, the Act grouped cannabis in with opioids and narcotics. This legislation has remained in effect since, and several presidents have campaigned on the promise of reducing drug use in the United States. Billions of US taxpayer dollars have been dedicated to condemnation of cannabis through media and education initiatives such as the DARE program [5]. At the same time the political landscape shifted towards anti-cannabis beliefs, the public opinion shifted to a more accepting and supportive stance [7].

The discrepancies between federal law and public opinion lead to a turbulent landscape for people seeking medicinal cannabis treatment. At the time of publication, 37 states, four U.S. territories, and the District of Columbia allow the medical use of cannabis [8]. However, patients have expressed concern that the stigma surrounding medical cannabis use would cause personal adverse impacts [9]. The stigma is substantial enough that many patients seek treatment from physicians they do not have a standing relationship with, decide not to seek treatment at all, or discontinue therapy [10]. Health education has been shown to be an effective measure in reducing negative stigma associated with medicinal cannabis use [11], but further analysis is needed to fully understand what demographic characteristics influence cannabis attitude to target education efforts.

This study aims to identify pertinent demographic characteristics. Including race, religion, political party, political ideologies, state legality, prior cannabis use, gender, marital status, age, ethnicity, employment status, and number of children, that have an effect on attitude toward cannabis. Due to the extensive history of political polarization of cannabis [12], demographic characteristics such as political ideologies and party affiliation were expected to significantly influence medical cannabis attitudes, while apolitical demographics were not predicted to have an impact on attitudes.

## 2 Methods

### 2.1 Scale selection

The Recreation and Medical Cannabis Scale [13] was used to measure participants' attitudes toward medical cannabis. The scale consists of two components: the Medical Cannabis Attitude Scale, with a reliability coefficient of 0.86, and the Recreational Cannabis Attitudes Scale, with a reliability coefficient of 0.91. This study only used the Medical Cannabis Attitude Scale. However, it was modified to remove the question asking “When I was 18, I believed that using marijuana for a medical purpose was acceptable,” as it was not displayed to anyone under the age of 35, to avoid confusing participants. The resulting scale, the Modified Medical Cannabis Attitude Scale (MMCAS) consisted of five Likert scales questions, with appropriate questions reverse coded, for a score range of five to 25. In addition to the validated questions from Arora et al. [13], demographic questions were asked of each respondent. The legal status of the respondents' states of residence was utilized in the analysis; to avoid inaccurate information impacting the analysis, respondents only reported their state of residence and the researchers coded each response based on the state's legal status at the time of data collection.

### 2.2 Survey administration

The survey was distributed via social media posts (paid advertisements and unpaid posts of the authors', university's, and research associates' accounts), including Facebook and Instagram, posted in the primary author's class learning management systems, and distributed to the university's lifelong learning database. Snowball sampling was also utilized. The survey was available from February 2021 to September 2021. Qualtrics was utilized for survey distribution and data collection. Once the survey was closed, responses from participants who did not complete the entire survey were eliminated from the analysis.

### 2.3 Data analysis

MMCAS scores were compared within demographic groups to determine factors that are associated with variable attitudes toward medical cannabis. A one-way ANOVA was used to determine differences within demographic characteristics. SPSS (v 27.0) was used to analyze the data. For groups that did not meet the assumption of homogeneity of variance, a one-way Welch ANOVA was run. A Tukey-Kramer, or Games-Howell for the one-way Welch ANOVA, post-hoc analysis was conducted to determine specific groups within the independent variables that significantly impacted the mean modified MCAS score differences [14,15].

### 2.4 Ethics

The Institutional Review Board at California State University Channel Islands (#IO5559) reviewed and approved the research protocols for this study. An electronic informed consent had to be acknowledged by respondents before they were able to access the survey. The raw data is not being made publicly available due to the sensitive nature of the questions and to be compliant with Institutional Review Board requirements.

## 3 Results

When the survey was closed, 673 participants had started the survey. Twenty-eight of the respondents did not complete the survey; that data was removed from the analysis. There were nine outliers noted, but were determined to be accurate numbers with minimal impact on the final means, therefore, they were not excluded. Data from the remaining 645 respondents was utilized for the analysis (Table 1).

**Table 1:** Respondent demographics.

	N	Percentage
Gender		
Male	154	23.9
Female	489	75.8
Non-binary	2	0.3

Age (years)		
18–24	198	30.7
25–34	113	17.5
35–44	123	19.1
45–54	88	13.6
55–84	74	11.5
65–74	32	5.0
75–84	14	2.2
85 or older	3	0.5

Ethnicity		
Hispanic	234	36.3
Non-Hispanic	411	63.7

Race		
White	392	60.8
Black or African American	52	8.10
Asian	42	6.51
American Indian or Alaska Native	4	0.6
Native Hawaiian or Pacific Islander	12	1.9
Other	143	22.2

Highest Degree		
Some High School	5	0.8
High School	234	36.3
Trade School	59	9.1
Bachelor's Degree	223	34.6
Master's Degree	89	13.8
Doctoral Degree	35	5.4

Employment Status		
Full time	313	48.5
Part time	98	15.2
Unemployed looking for work	31	4.8
Unemployed not looking for work	33	5.1
Retired	73	11.3
Student	77	11.9
Disabled	20	3.1

Political Party Affiliation		
Democratic	325	50.4
Republican	114	17.7
Independent / No party affiliation	141	21.9
Libertarian	21	3.3
Other	14	2.2
Not registered	30	4.7

Political View		
Very liberal	122	18.9
Slightly liberal	161	25.0
Moderate	257	39.8
Slightly conservative	77	11.9
Very conservative	28	4.30

State Legal Status		
Illegal	45	6.97
Medicinal only	71	11.0
Medicinal & recreational	529	82.0

Religion		
Catholicism/ Christianity	384	59.5
Judaism	13	2.0
Islam	7	1.1
Buddhism	9	1.4
Hinduism	1	0.2
Other religion	54	8.4
No religion	177	27.4

The MMCAS scores were not normally distributed for the groups of independent variables, as assessed by Shapiro-Wilk's test (*p* < 0.05), and visual inspection of Q-Q plots. Given that ANOVA is robust deviations from normality, the parametric test was still appropriate [16,17].

Significant variation in MMCAS was noted between groups based on race, political party affiliation, political view, religion, state legal status, and past/current cannabis use. There were no significant variations noted in MMCAS based on gender, age, ethnicity, education, marital status, number of children, or employment status (Table 2). The specifics of each of these are explained below.

**Table 2:** Mean MCAS scores based on demographic variables.

	N	MCAS	*p* value
Overall Mean	645	20.0	

Gender			0.360
Male	154	20.1	
Female	489	19.94	
Non-binary	2	16.0	

Age (years)			0.095
18–24	198	19.4	
25–34	113	20.6	
35–44	123	20.4	
45–54	88	20.0	
55–64	74	20.4	
65–74	32	19.6	
75–84	14	19.5	
85 or older	3	14.3	

Ethnicity			0.052
Hispanic	234	19.6	
Non-Hispanic	411	20.2	

Race			0.024
White	392	19.4	
Black or African American	52	20.1	
Asian	42	18.3	
American Indian or Alaska Native	4	21.5	
Native Hawaiian or Pacific Islander	12	19.8	
Other	143	20.3	

Marital Status			0.511
Married	266	20.0	
Never married	291	19.9	
Divorced	62	20.7	
Separated	5	20.8	
Widowed	21	18.9	

Number of Children			0.398
0	318	20.1	
1	95	20.0	
2	133	20.3	
3 or more	99	19.2	

Highest Degree			0.417
Some High School	5	20.0	
High School	234	19.6	
Trade School	59	20.3	
Bachelor's Degree	223	20.4	
Master's Degree	89	19.8	
Doctoral Degree	35	19.5	

Employment Status			0.112
Full time	313	20.1	
Part time	98	19.3	
Unemployed looking for work	31	19.6	
Unemployed not looking for work	33	21.3	
Retired	73	20.7	
Student	77	19.4	
Disabled	20	19.7	

Political Party Affiliation			0.011
Democratic	325	20.4	
Republican	114	18.9	
Independent / No party affiliation	141	19.9	
Libertarian	21	20.4	
Other	14	20.7	
Not registered	30	18.7	

Political View			<0.0005
Very liberal	122	22.0	
Slightly liberal	161	20.3	
Moderate	257	19.4	
Slightly conservative	77	18.9	
Very conservative	28	17.8	

State Legal Status			0.010
Illegal	45	20.1	
Medicinal only	71	21.4	
Medicinal & recreational	529	19.8	

Religion			0.004
Catholicism/ Christianity	384	19.5	
Judaism	13	19.6	
Islam	7	16.7	
Buddhism	9	19.2	
Other religion	54	20.6	
No religion	177	21.1	
*Hinduism*	*1*	*23.0*	*Excluded from religion analysis*

Cannabis Use			<0.0005
Current or past use	466	20.9	
Never	179	17.6	

### 3.1 Significant results

#### 3.1.1 Race

**Fig. 1:**
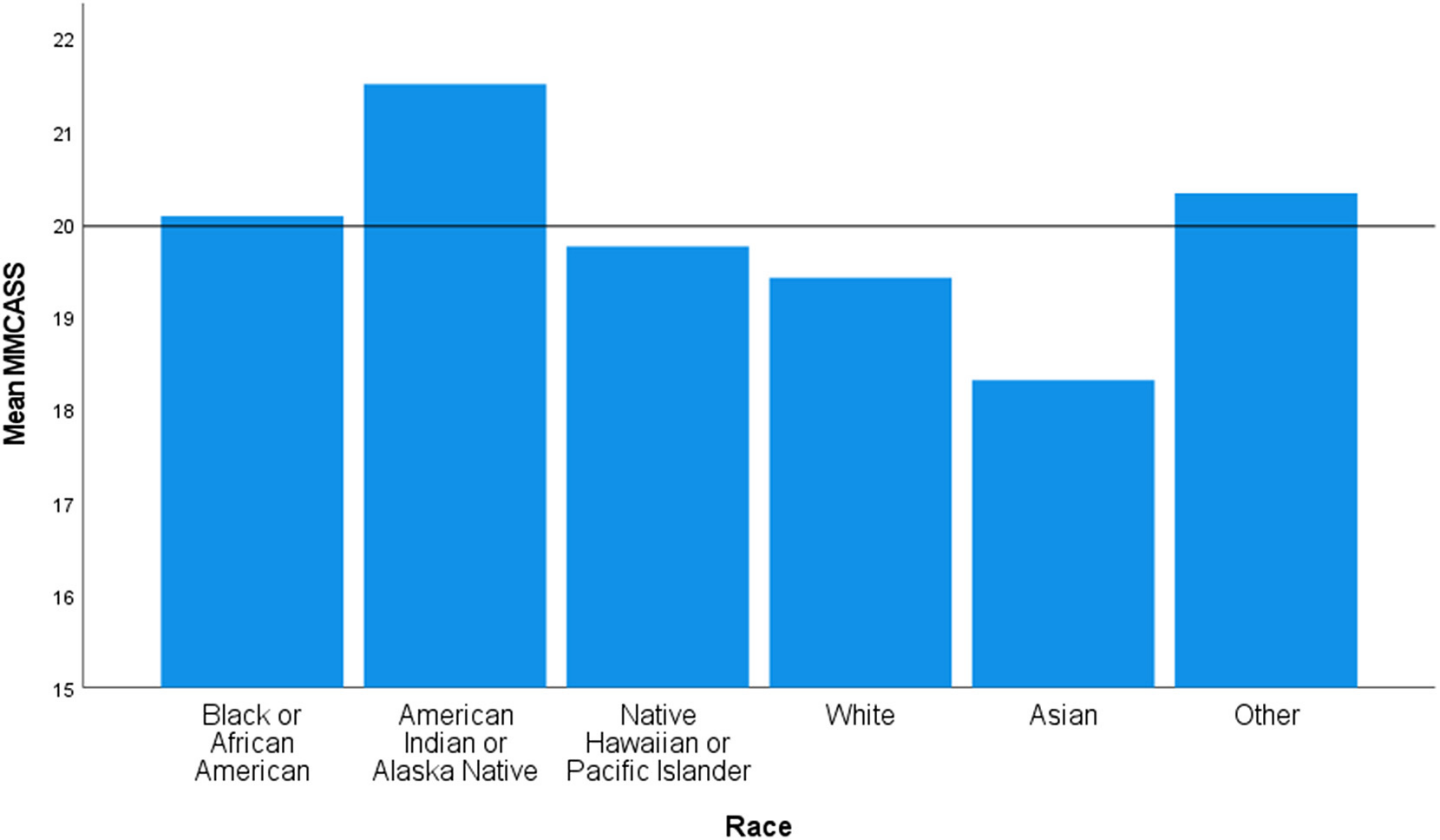
Mean modified medical cannabis attitude scale score for different race categories.

Levene's test for equality of variance showed the assumption of homogeneity was met for race (*p* = 0.098). The mean modified MCAS scores varied significantly between self-identified racial groups, *F*(5, 639) = 2.60, *p* = 0.024 (Fig. 1). Tukey-Kramer post hoc analysis revealed a higher MMCASS in those reporting their race as Other compared to those reporting as Asian (2.01, 95% CI (0.11 to 3.92)), a statistically significant difference (*p* = 0.032), but no other group differences were statistically significant.

#### 3.1.2 Political party

**Fig. 2:**
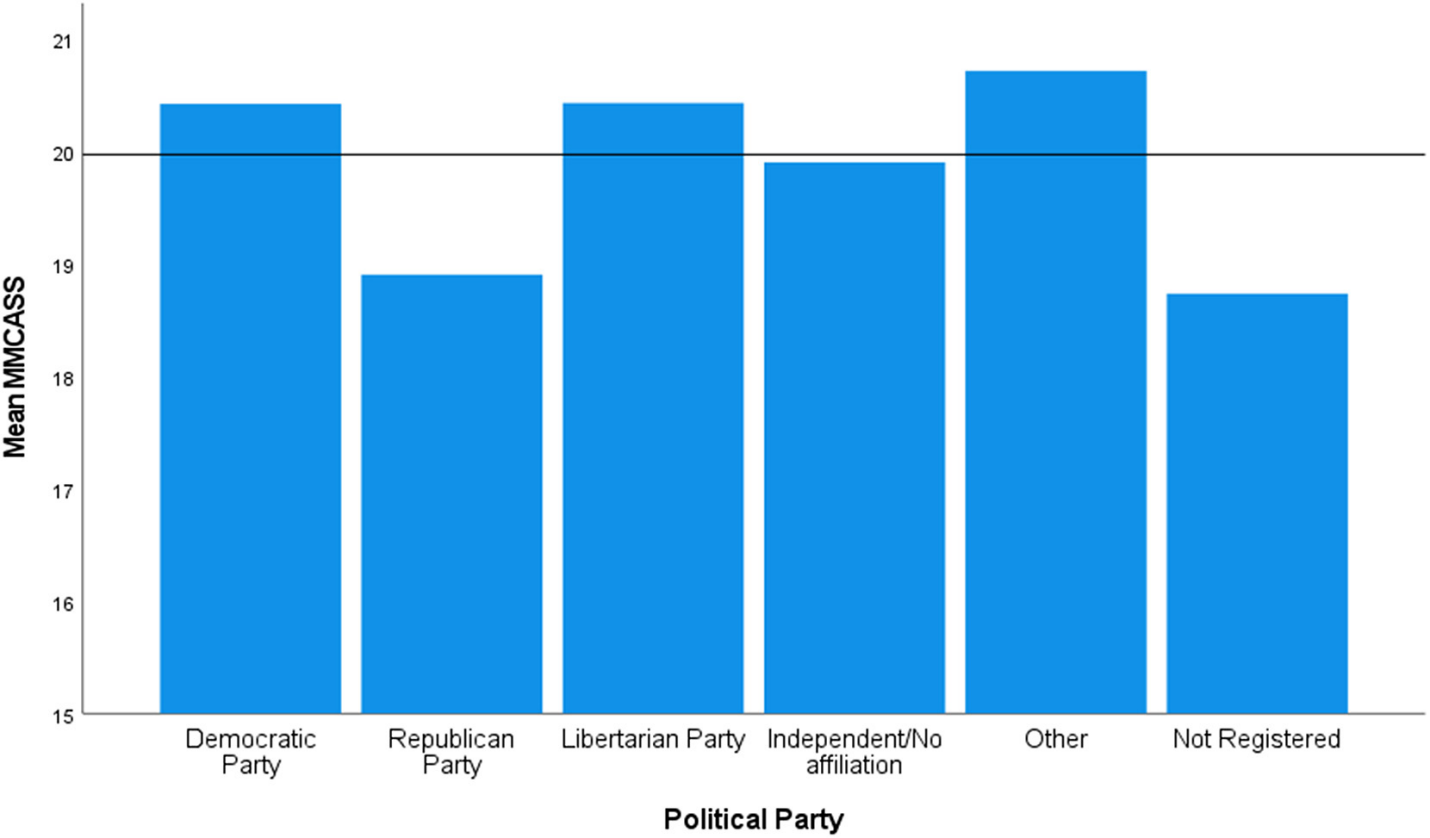
Mean modified medical cannabis attitude scale score for political party affiliation.

The assumption of homogeneity of variances was met for political party affiliation, as assessed by Levene's test for equality of variances (*p* = 0.335). The modified MCAS score was significantly different based on party affiliation, *F*(5, 639) = 3.02, *p* = 0.011 (Fig. 2). A statistically significant higher MMCASS for Democrat participants compared to Republican (*p* = 0.009) was confirmed by the Tukey-Kramer post hoc analysis (1.518, 95% CI (0.24 to 2.80)). No other group differences were statistically significant. Due to the markedly lower MMCAS scores in those not registered to vote compared to each political party affiliation option, another ANOVA was run comparing those not registered to vote to all other respondents (Fig. 2). No statistical significance was found with voter registration status, *F*(1, 643) = 2.81, *p* = 0.094.

#### 3.1.3 Political view

**Fig. 3:**
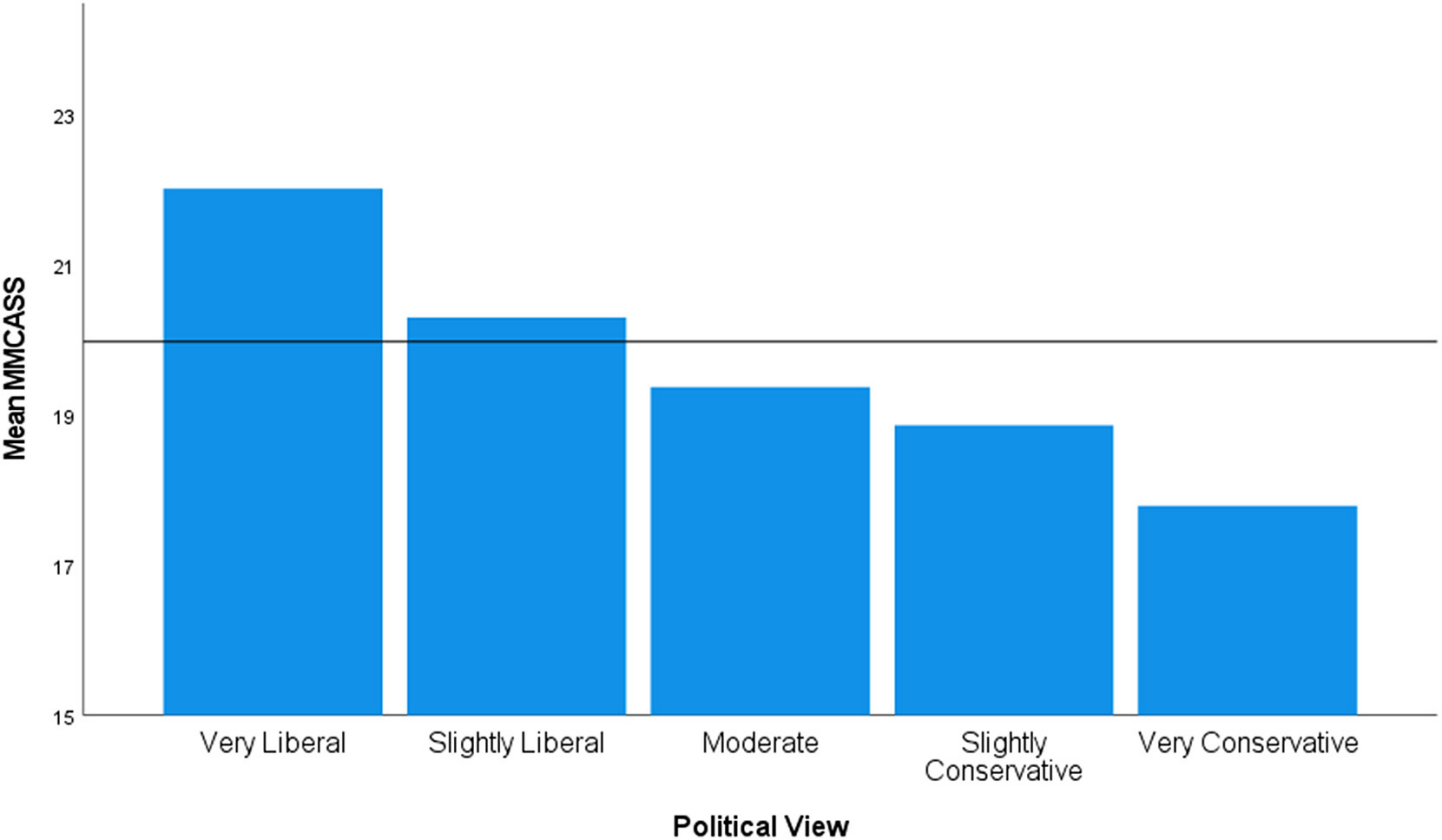
Mean modified medical cannabis attitude scale score for political view.

For political view, the assumption of homogeneity of variances was violated, as assessed by Levene's test for equality of variances (*p* < 0.0005). There were statistically significant differences in modified MCAS score between groups, Welch's *F*(4, 145.273) = 16.1, *p* < 0.0005 (Fig. 3). Survey respondents reporting their political view as “very liberal” had a statistically significant higher MMCASS than every other view, as revealed by the Games-Howell post hoc analysis:

• Vs. slightly liberal (1.72, 95% CI (0.60 to 2.83), *p* < 0.0005)
• Vs. moderate (2.64, 95% CI (0.158 to 3.70), *p* < 0.0005)
• Vs. slightly conservative (3.15, 95% CI (1.42 to 4.88), *p* < 0.0005)
• Vs. very conservative (4.22, 95% CI (1.74 to 6.71), *p* < 0.0005)

#### 3.1.4 Religion

**Fig. 4:**
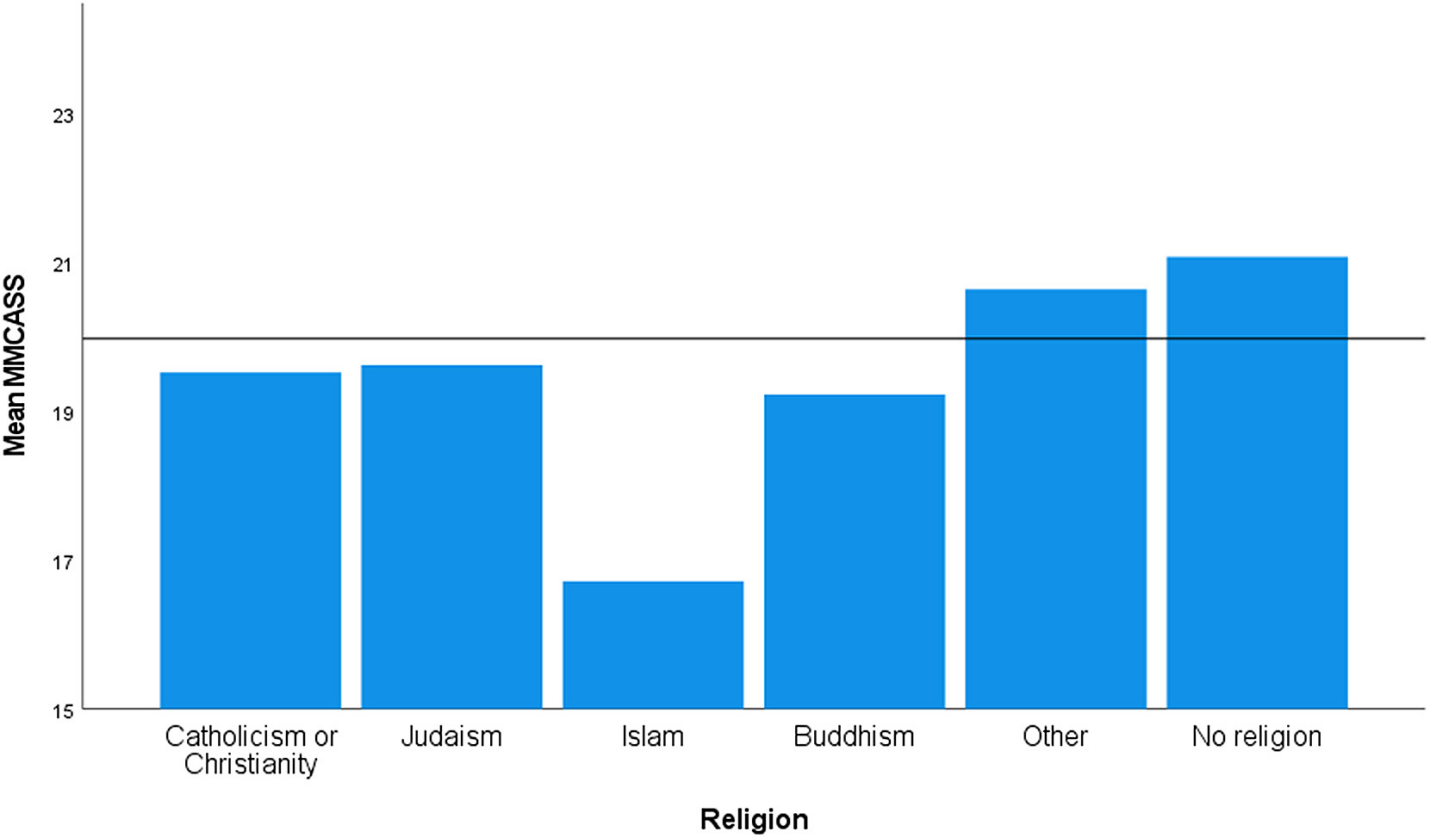
Mean modified medical cannabis attitude scale score for religion.

For the data on religion, Levene's test for equality of variance showed the assumption of homogeneity was not met (*p* = 0.039). The one participant who reported their religion as Hindu had to be removed from the religion analysis because tests of equality means cannot be performed when one group has less than two cases. The differences in mean MMCASS were significant, Welch's *F*(5, 28.954) = 4.50, *p* = 0.004 (Fig. 4). The Games-Howell post hoc analysis revealed a higher MMCASS in those reporting no religious affiliation compared to those reporting being Catholic/Christian (1.55, 95% CI (0.58 to 2.52)), a statistically significant difference (*p* < 0.05), but no other group differences were statistically significant. The mean MMCASS for those reporting as Islamic was 16.71 compared to a mean of all participants of 20.0; the next lowest mean of 19.2 was for those who were Buddhist. However, due to the proportion of the sample who reported to be Islamic, this was not a statistically significant result.

#### 3.1.5 State legal status

**Fig. 5:**
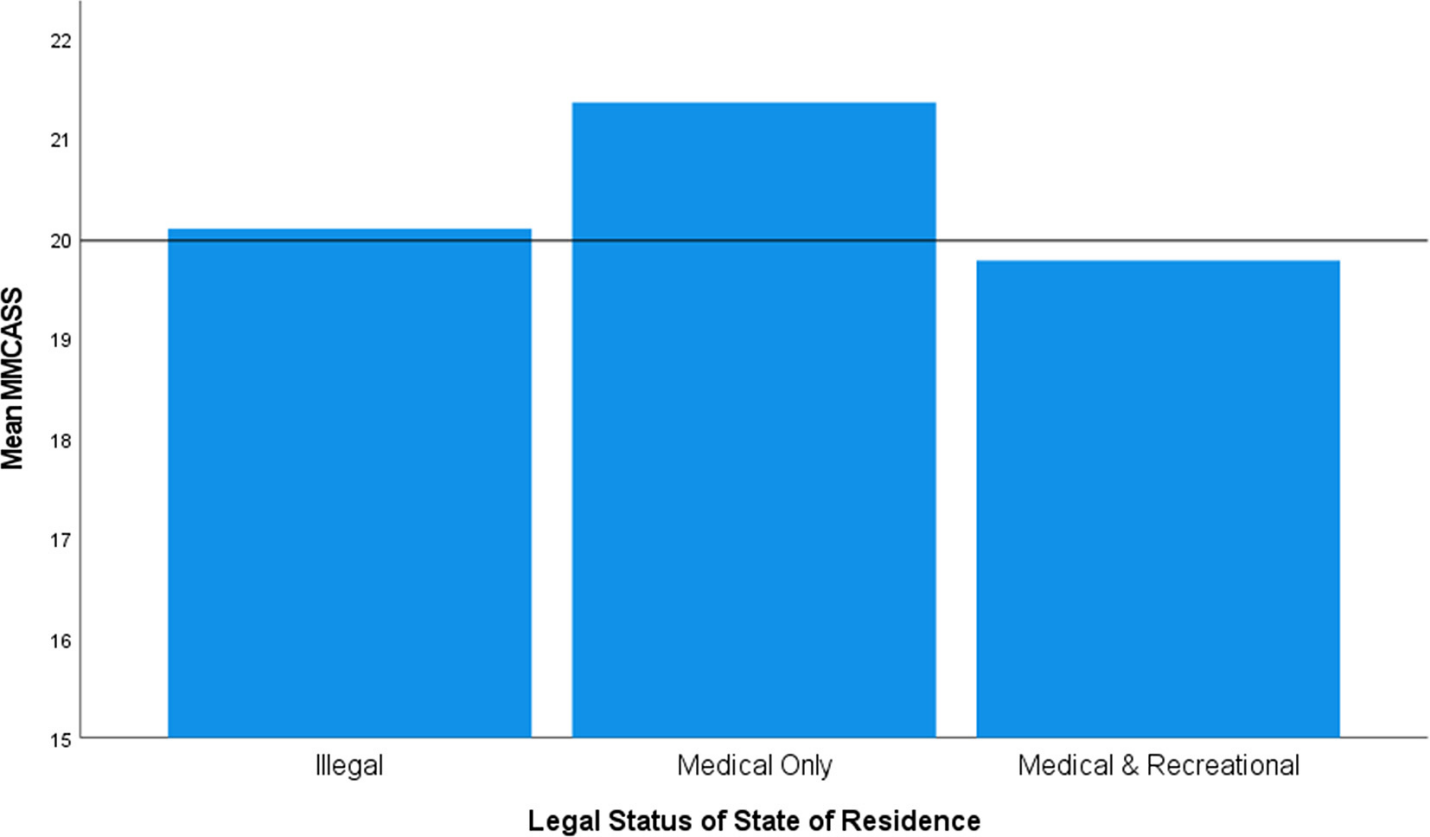
Mean modified medical cannabis attitude scale score for respondents based on the cannabis legal status of their state of residence.

The assumption of homogeneity of variances was met, as assessed by Levene's test for equality of variances (*p* = 0.188). The modified MCAS score was significantly different based on the legal status of cannabis in the participants' states of residence, *F*(2, 642) = 4.639, *p* = 0.010 (Fig. 5). The Tukey-Kramer post hoc analysis uncovered a statistically significant higher MMCASS for respondents who lived in a state with medical only access to cannabis compared to those with medical and recreation access (*p* = 0.007) was revealed (1.60, 95% CI (0.36 to 2.80)). No other group differences were statistically significant.

#### 3.1.6 Cannabis use

**Fig. 6:**
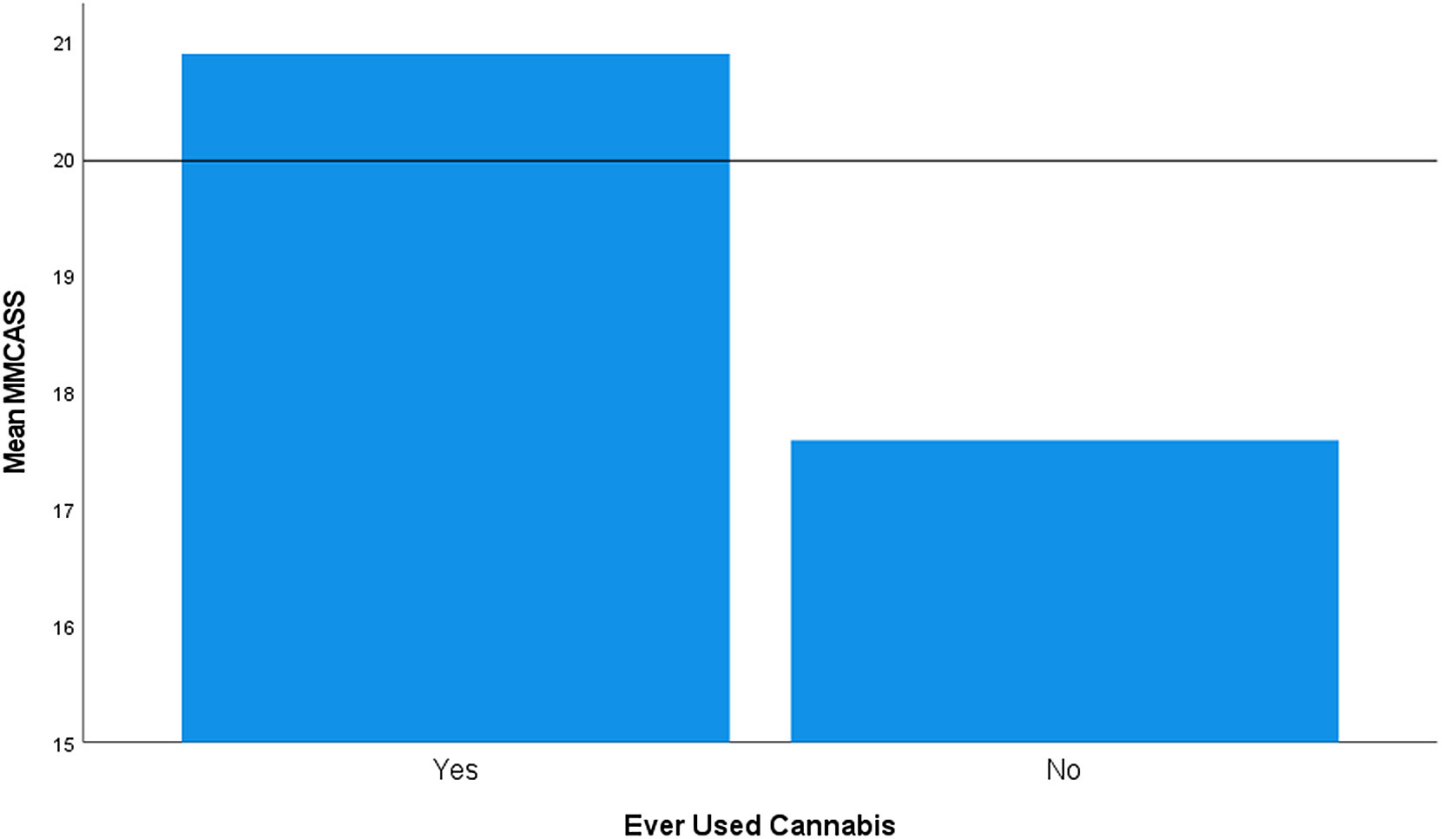
Mean modified medical cannabis attitude scale score of participants grouped by their past/current cannabis use.

The assumption of homogeneity of variances was met, as assessed by Levene's test for equality of variances (*p* = 0.051) (Fig. 6). The modified MCAS score was significantly different for cannabis use, *F*(1, 643) = 94.298, *p* < 0.0005.

### 3.2 Non-significant results

#### 3.2.1 Gender

The assumption of homogeneity of variances was met, as assessed by Levene's test for equality of variances (*p* = 0.625). The modified MCAS score was not significantly different for gender, *F*(2, 642) = 1.023, *p* = 0.360.

#### 3.2.2 Age

The assumption of homogeneity of variances was violated, as assessed by Levene's test for equality of variances (*p* = 0.034). There were no statistically significant differences in modified MCAS score between age groups, Welch's *F*(7, 33.037) = 1.937, *p* = 0.095.

#### 3.2.3 Ethnicity

Levene's test for equality of variance showed the assumption of homogeneity was met (*p* = 0.405). The differences in mean modified MCAS scores between those identifying as Hispanic and non-Hispanic did not vary significantly, *F*(1, 643) = 3.806, *p* = 0.052.

#### 3.2.4 Education

Levene's test for equality of variances revealed the data did not meet the assumption of homogeneity (*p* = 0.001). The mean modified MCAS scores between different education levels did not vary significantly, Welch's *F*(5, 38.845) = 1.024, *p* = 0.417.

#### 3.2.5 Marital status

The assumption of homogeneity of variances was violated, as assessed by Levene's test for equality of variances (*p* < 0.0005). There were no statistically significant differences in modified MCAS score based on marital status, Welch's *F*(4, 25.836) = 0.843, *p* = 0.511.

#### 3.2.6 Number of children

Levene's test for equality of variances showed the data violated the assumption of homogeneity (*p* = 0.002). The mean modified MCAS scores did not vary significantly regardless of the number of children a participant reported, Welch's *F*(3, 232.789) = 0.990, *p* = 0.398.

#### 3.2.7 Employment status

The assumption of homogeneity of variances was met with employment status, as assessed by Levene's test for equality of variances (*p* = 0.123). The modified MCAS score was not significantly different, *F*(6, 638) = 1.727, *p* = 0.112.

## 4 Discussion

### 4.1 Discussion

The shifting opinions toward medical cannabis over the past several decades has led to more states legalizing the plant and improving patient access to it [18,19]. Nonetheless, patients still report issues with obtaining and utilizing medical cannabis because of lingering stigma towards it [9,10]. Understanding the demographic factors that are associated with groups of people having negative views toward medical cannabis will help advocates and public health officials focus education efforts.

The results of this analysis were consistent with the hypothesis. There were significant differences in attitudes toward medical cannabis between racial groups, political party affiliation, political view, religion, state legal status, and cannabis use. The demographic variables of gender, age, ethnicity, education level, marital status, number of children, and employment status did not yield any significant variations between groups. The former all include political, religious, or legal factors while the latter do not.

#### 4.1.1 Demographic factors associated with differences in medical cannabis attitudes

The only significant difference noted between different racial groups was those self-identified as Asian compared to those who self-identified as Other. Asians had a numerically lower MMCAS score versus every other racial category. Anecdotally, several of the participants who self-identified as Asian took the extra step to contact the primary investigator after completing the informed consent and survey to further express their disapproval of medical cannabis. American Indian and Alaska Native respondents had a markedly higher MMCAS score despite the differences between every other racial group not being significant; this is likely due to the low number of respondents self-reporting as American Indian or Alaska Native. The positive attitudes seen in this group is likely related to the cultural and historical uses of cannabis and other similar herbs in ceremonial practices [20,21].

Republican respondents being less favorable toward medical cannabis as compared to Democratic respondents is consistent with other research [7]. Independents and Democrats have been more likely to support legalization of cannabis since the early 1990s [[22], [23], [24]]. Those who indicated they are not registered to vote had a noticeably lower MMCAS score, but was not statistically significant versus the other groups because of the small number of respondents indicating such. Previous research has identified similar attitude variations between political affiliations, but has not investigated differences between registered voters and non-registered voters [25].

There was an inverse relationship between political views and attitudes toward medical cannabis. As one's level of conservativeness increased, they were more likely to have unfavorable attitudes toward the plant (Fig. 3). This is consistent with what has been observed in political support for the War on Drugs and other prohibition efforts [5,12].

One's religion can have an impact on attitudes toward medical cannabis. Catholic/Christian respondents had a significantly more negative view toward medical cannabis than those who were not religious. With the exception of those who selected “Other” as their religion, all religious groups were numerically less than those who had no religion. There were other variations with those who are Jewish, Buddhist, or particularly Islamic having noticeably unfavorable attitudes toward medical cannabis; these individual faiths, though, did not have a sufficient number of respondents to reach statistical significance. This is similar to the findings Edelstein et al. [26] reported that religious college students report using cannabis less often, having less contact with individuals who use cannabis, and being less likely to support cannabis legalization (recreational or medicinal).

In terms of the legal status of the respondents' states of residence, the most favorable views toward medical cannabis were seen from those who live in states with medical-access only. These views were significantly different from those who live in states with access to both medical and recreational use. The more negative views with recreational legalization is thought to be related to some of the unsavory elements of allowing recreational use: increased traffic accidents with the driver testing positive for cannabis, higher hospitalization rates, complaints about odors permeating into private residences, and increased referrals in public schools for cannabis use on campus [[27], [28], [29]].

There were significantly more positive views toward medical cannabis from those who had used it previously than from those who had never used it. This is likely related to the long-term villainization of the plant by the U.S. Federal government and other influential entities beginning in the early 1900s [1,3,5].

#### 4.1.2 Demographic factors not associated with differences in medical cannabis attitudes

All of the demographic factors that did not include political, religious, or legal undertones were not associated with any differences in attitudes toward medical cannabis. This speaks to two phenomena: (1) the increasing approval of medical cannabis [1] and (2) the potential application of medical cannabis to people affected by a wide range of medical ailments [[30], [31], [32]]. Given that prior use is associated with more positive attitudes, when controlling for political, religious, and legal factors, it would be expected that other demographic factors would not contribute further to either positive or negative attitudes.

#### 4.1.3 Limitations

The present research was limited by the high number of respondents who identified as White, female, current or previous users of cannabis, and living in a state with recreational access to cannabis. There were also small numbers of respondents identifying as American Indian or Alaska Native, Islamic, or Hindu. Given that the mean MMCAS scores for these groups deviated from the overall mean to a notable degree, it would be interesting to determine if the results would hold consistent with a larger representation.

### 4.2 Innovation

Some previous research has focused on either cannabis in general, the legalization of cannabis, or the legalization of medical cannabis [32]. Other research has examined patient attitudes toward medical cannabis, but not using a validated scale [[33], [34]]. This current research is the analysis of demographic factors that impact attitudes toward medical cannabis. This was innovative in that in (1) utilized a validated scale measuring attitudes toward medical cannabis, (2) analyzed more complex attitudes rather than simply support for legalization of the plant, (3) focused on medical use of cannabis instead of generalization of cannabis regardless of reasons behind its use, and (4) evaluated attitudes from the general population, not specifically patients [13].

This study clearly identified factors that impact attitudes toward medical cannabis. Previous research has also confirmed that health education efforts can improve attitudes toward it [11]. Cannabis advocates can innovatively apply health education efforts to those groups of people who are aligned with the demographic factors identified in this current work.

### 4.3 Conclusion

There is a need to improve patient access to medical cannabis and reduce stigmas associated with its use. This survey of 645 individuals from across the United States identified factors that influence said attitudes. The use of health education targeted at the groups of people who continue to harbor antiquated attitudes toward medical cannabis will help to improve patient access and, thus, patient outcomes.

## Declaration of Competing Interest

The authors declare that they have no known competing financial interests or personal relationships that could have appeared to influence the work reported in this paper. There was no external funding received for this research.
